# CCR3 deficiency is associated with increased osteoclast activity and reduced cortical bone volume in adult male mice

**DOI:** 10.1074/jbc.RA120.015571

**Published:** 2020-12-17

**Authors:** Sara Rosendahl, Rima Sulniute, Michaela Eklund, Cecilia Koskinen Holm, Marcus J.O. Johansson, Elin Kindstedt, Susanne Lindquist, Pernilla Lundberg

**Affiliations:** 1 Department of Odontology, Section of Molecular Periodontology, Umeå University, Umeå, Sweden; 2 Wallenberg Centre for Molecular Medicine, Umeå University, Umeå, Sweden

**Keywords:** chemokine, receptor, osteoclast, osteoblast, bone, gene knockout, μCT, microcomputed tomography, α-MEM, minimum essential medium Eagle, alpha modification, BFR, bone formation rate, BMM, bone marrow macrophage, BS, bone surface, CCL, C–C motif chemokine ligand, CCR3, C–C motif chemokine receptor 3, CTX-I, C-terminal type I collagen, M-CSF, macrophage colony-stimulating factor, MAR, mineral apposition rate, MS, mineralizing surface, qPCR, quantitative PCR, RA, rheumatoid arthritis, RANKL, receptor activator of nuclear factor kappa-B ligand, TRAP5b, tartrate-resistant acid phosphatase 5b

## Abstract

Increasing evidence emphasizes the importance of chemokines and chemokine receptors as regulators of bone remodeling. The C–C chemokine receptor 3 (CCR3) is dramatically upregulated during osteoclastogenesis, but the role of CCR3 in osteoclast formation and bone remodeling in adult mice is unknown. Herein, we used bone marrow macrophages derived from adult male CCR3-proficient and CCR3-deficient mice to study the role of CCR3 in osteoclast formation and activity. CCR3 deficiency was associated with formation of giant hypernucleated osteoclasts, enhanced bone resorption when cultured on bone slices, and altered mRNA expression of related chemokine receptors and ligands. In addition, primary mouse calvarial osteoblasts isolated from CCR3-deficient mice showed increased mRNA expression of the osteoclast activator–related gene, receptor activator of nuclear factor kappa-B ligand, and osteoblast differentiation–associated genes. Microcomputed tomography analyses of femurs from CCR3-deficient mice revealed a bone phenotype that entailed less cortical thickness and volume. Consistent with our *in vitro* studies, the total number of osteoclasts did not differ between the genotypes *in vivo*. Moreover, an increased endocortical osteoid mineralization rate and higher trabecular and cortical bone formation rate was displayed in CCR3-deficient mice. Collectively, our data show that CCR3 deficiency influences osteoblast and osteoclast differentiation and that it is associated with thinner cortical bone in adult male mice.

During physiological bone remodeling, the actions of bone-forming osteoblasts and bone-resorbing osteoclasts cooperate in order to successively rebuild the skeleton, a highly balanced process that is crucial to preserve normal skeletal functions and to maintain bone mass. Osteoblasts and osteocytes express receptor activator of nuclear factor kappa-B ligand (RANKL), the key protein that promotes differentiation of cells of the macrophage/monocyte lineage into mature osteoclasts with bone resorptive capacity. In addition to hormones and mechanical loading, molecules that are primarily associated with the immune system, *e.g.*, cytokines and chemokines, regulate the level of available RANKL and thus also control the bone remodeling process (1). Inflammation may disturb the prevailing equilibrium between bone formation and bone resorption, which most often manifest as a net bone loss because of excessive formation and activity of osteoclasts. Pathological bone loss is a hallmark of chronic inflammatory conditions, such as rheumatoid arthritis (RA), periodontitis, and loosened joint prosthesis and tooth implants (1).

Chemokines are key regulators of the innate and adaptive immune system. They are released by a wide variety of cell types in response to infection or tissue damage and form chemotactic gradients that attract leukocytes to sites of inflammation, which further facilitate the immune response by mediating angiogenesis (2). Besides the involvement of chemokines during pathological conditions, chemokines are also involved in homing of immune cells to lymph nodes during homeostasis. The mode of action relies on interaction between chemokines and their cognate receptors, a system that is highly complex as there are approximately 50 chemokine ligands and 20 chemokine receptors known to date (3). Hence, multiple ligands share the same receptor. Although the crossreactivity is comprehensive, most chemokines have a preferred high-affinity receptor (4).

Increasing evidence shows that chemokine signaling promotes crosstalk between the cells of the immune system and bone cells and thus plays a crucial role in the bone remodeling process during physiological and pathological conditions (5). We have previously shown that individuals with periodontitis, a condition that entails jawbone loss triggered by chronic inflammation in the gum, present with systemically and locally higher levels of the C–C motif chemokine ligand 11 (CCL11) (6). Further experimental studies revealed that CCL11 is highly expressed by osteoblasts during inflammation-induced bone resorption (7). In line with our findings, others have shown high CCL11 levels in conditions that are characterized by pathological bone loss, *e.g.*, RA (8), osteoarthritis (9), and osteoporosis (10), strongly suggesting that CCL11 plays a decisive role in inflammation-induced bone resorption. CCL11 binds to the chemokine receptors CCR2, CCR3, and CCR5, with highest affinity to CCR3 (11, 12).

The importance of CCR2 and CCR5 signaling in the continuous balancing of bone mass and during osteoclastogenesis has been demonstrated in chemokine and chemokine receptor deficiency *in vivo* models. Mice lacking either the receptor CCR2 or its primary ligand CCL2 display a reduction in osteoclast numbers and size, resulting in decreased bone resorption and severe osteopetrosis (13, 14). Mice lacking CCR5 are resistant to RANKL-induced bone loss, and osteoclasts from CCR5-deficient mice show defect adhesion, reduced locomotion, and resorptive capacity (15). Together, these results indicate essential roles for CCR2, CCR5, and their associated ligands in bone-destructive conditions through the influence on osteoclast function.

Despite the fact that CCR3 expression is highly upregulated during osteoclastogenesis, the importance of CCR3 signaling in normal bone remodeling and pathological conditions affecting the bone remains largely unknown. CCR3 can bind several ligands, of which CCL7 stimulates osteoclast differentiation *in vitro* (16) and CCL5 (also known as regulated on activation, normal T cell expressed, and secreted, RANTES) is chemotactic for osteoblasts and has an inhibitory effect on osteoclasts (17, 18). Interestingly, its most high-affinity ligand CCL11 is reported to increase preosteoclast migration and osteoclastic bone resorption (7, 17, 18).

Herein, we study the effect of CCR3 deficiency on osteoclast differentiation, bone resorption, and osteoblast gene expression *in vitro*. Furthermore, we analyze the bone phenotype of CCR3-deficient adult male mice.

## Results

### CCR3 deficiency results in increased osteoclast size and number of nuclei per osteoclast

**Figure 1:**
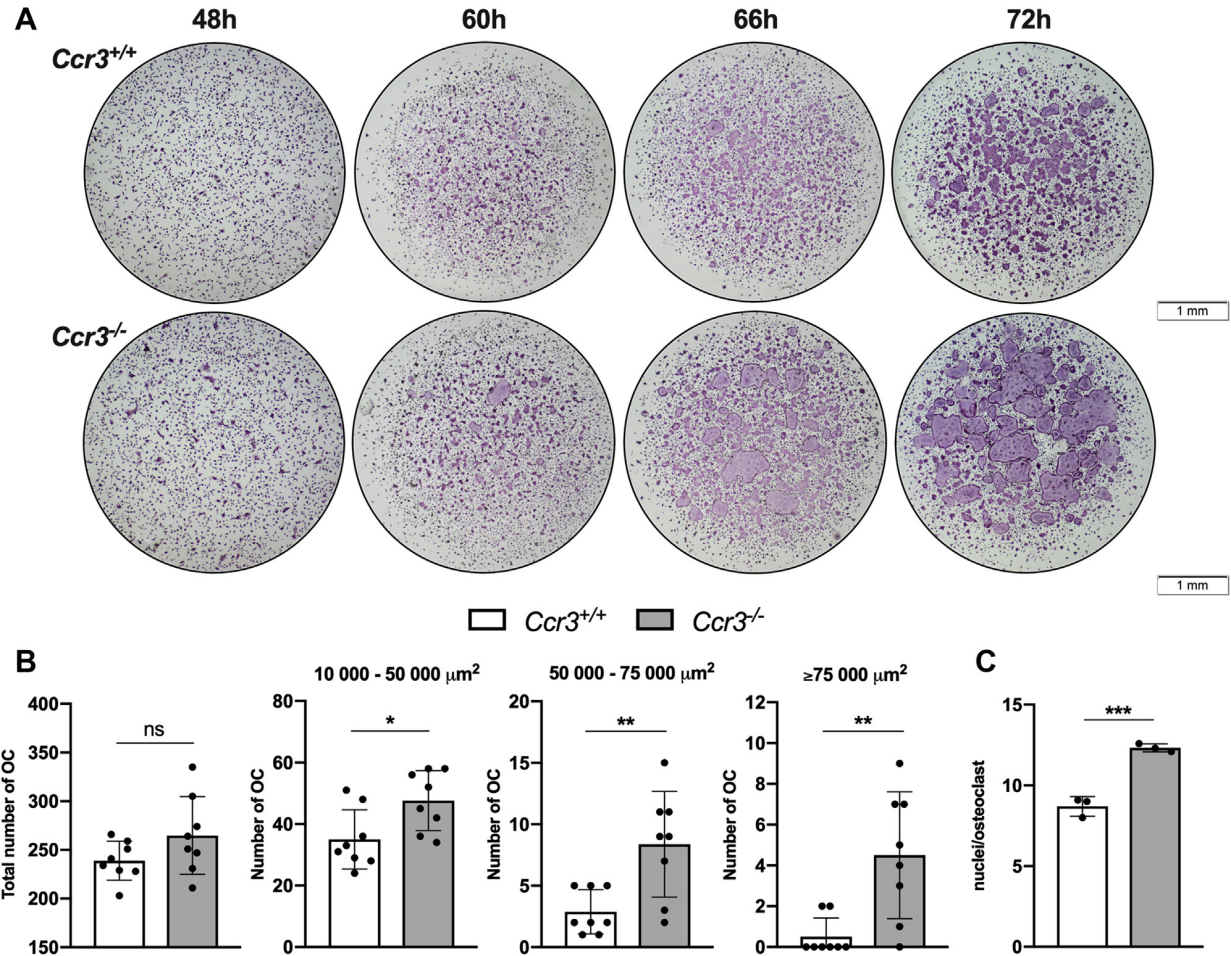
**Increased osteoclast (OC) size and number of nuclei in *Ccr3***^***−/−***^**bone marrow macrophage (BMM) cultures.** Mouse BMMs derived from C–C chemokine receptor 3 (CCR3)-deficient (*Ccr3*^*−/−*^) and wildtype (*Ccr3*^*+/+*^) mice were cultured on plastic, supplemented with macrophage colony-stimulating factor and receptor activator of nuclear factor kappa-B ligand and stained for tartrate-resistant acid phosphatase. *A*, *Ccr3*^*−/−*^ BMMs developed into significantly larger OCs compared with *Ccr3*^*+/+*^ BMMs. Representative images are shown. *B*, the number and size of *Ccr3*^*−/−*^ and *Ccr3*^*+/+*^ OCs was measured after 72 h incubation. There was no significant difference in total OC numbers. However, when the cells >10,000 μm^2^ were stratified in three different size groups, the number of *Ccr3*^*−/−*^ OCs was significantly higher in all groups compared with *Ccr3*^*+/+*^ OCs, n = 8 wells per genotype. *C*, quantification of number of nuclei per OC in *Ccr3*^*−/−*^ and *Ccr3*^*+/+*^ cultures shows significantly greater number of nuclei in *Ccr3*^*−/−*^ OCs, n = 3 wells per genotype. Data represent mean values ± SD. ∗*p* < 0.05; ∗∗*p* < 0.01; and ∗∗∗*p* < 0.001.

To study if CCR3 deficiency affects osteoclastogenesis and osteoclast morphology, we cultured primary bone marrow macrophages (BMMs) from *Ccr3*^*−/−*^ and *Ccr3*^*+/+*^ mice in the presence of macrophage colony-stimulating factor (M-CSF) and RANKL. The osteoclasts formed after 48 h tended to be larger in size in the *Ccr3*^*−/−*^ than in the *Ccr3*^*+/+*^ cultures. This size difference was even more pronounced after 60 and 66 h of culture. After 72 h, *Ccr3*^*−/−*^ osteoclasts were highly oversized (Fig. 1*A*), which also could be verified quantitatively as the number of large osteoclasts (>10,000 μm^2^) was significantly higher in the *Ccr3*^*−/−*^ osteoclast cultures (1.4-fold to 9-fold; Fig. 1*B*). Importantly, the *Ccr3*^*+/+*^ osteoclasts did never reach the same giant size as *Ccr3*^*−/−*^ osteoclasts, even though the culture time was extended (96 h; Fig. S1). Moreover, the number of nuclei per osteoclast was higher in *Ccr3*^*−/−*^ osteoclast cultures (Fig. 1*C*). However, the total number of osteoclasts did not differ between the genotypes after 48 h or after 72 h of culture (Fig. 1*B* shows number of osteoclasts at 72 h). To analyze if even heterozygous osteoclasts present with an increase in cell size, osteoclasts derived from BMMs were cultured from *Ccr3*^*−/−*^, *Ccr3*^*+/−*^, and *Ccr3*^*+/+*^ mice. The results show that oversized osteoclasts could be seen also in the *Ccr3*^*+/−*^ cultures, although not as marked as in cells from the homozygous phenotype (Fig. S1).

### CCR3-deficient osteoclasts cultured on bone slices show increased size and bone resorption

**Figure 2:**
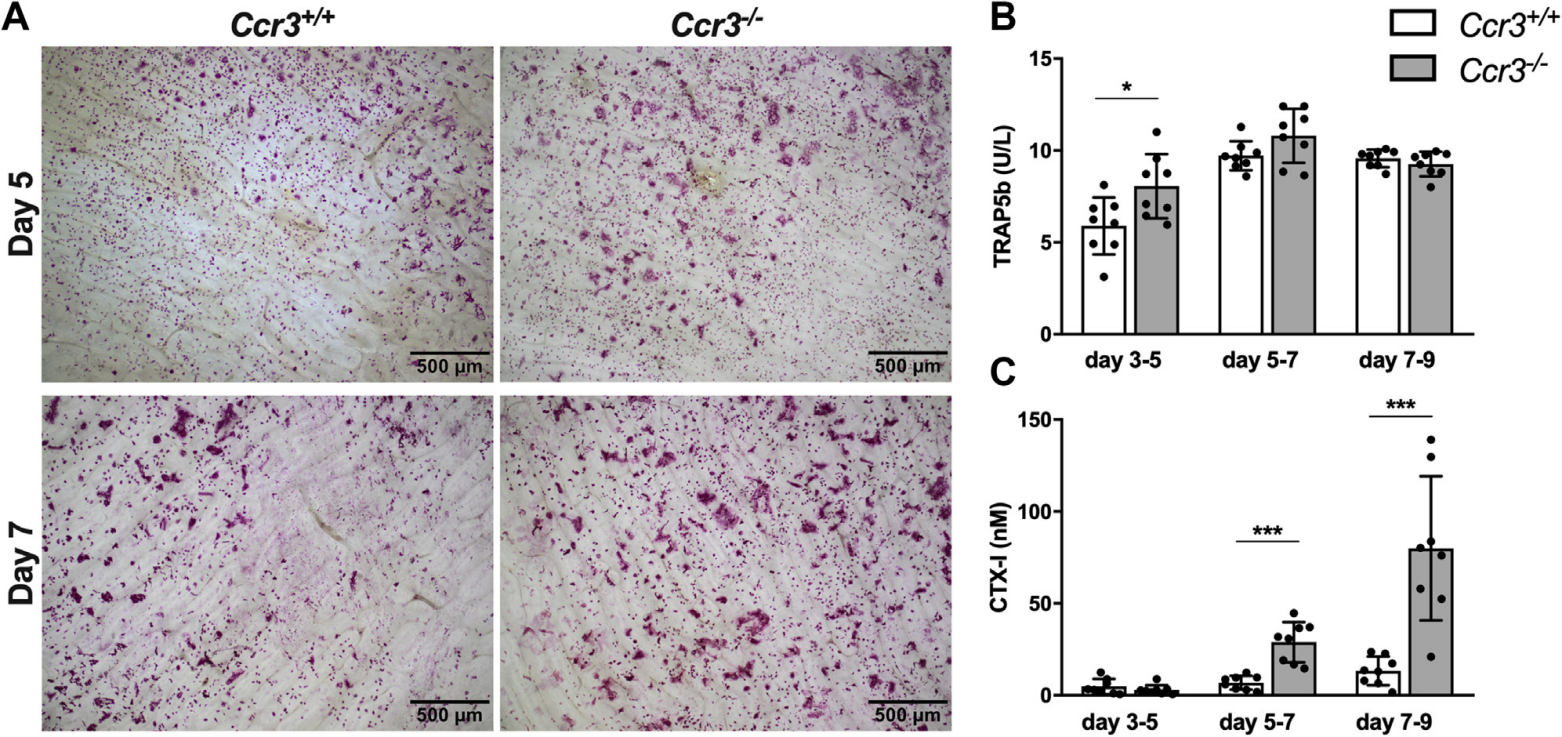
**Increased osteoclast size, TRAP5b release, and bone resorption in *Ccr3***^***−/−***^**osteoclasts cultured on bone slices.** Mouse bone marrow macrophages (BMMs) derived from *Ccr3*^*−/−*^ and *Ccr3*^*+/+*^ were cultured with macrophage colony-stimulating factor and receptor activator of nuclear factor kappa-B ligand for 9 days. *A*, BMM-derived osteoclasts from *Ccr3*^*−/−*^ and *Ccr3*^*+/+*^ mice stained for TRAP. *Ccr3*^*−/−*^ BMMs developed into significantly larger osteoclasts compared with *Ccr3*^*+/+*^ BMMs. Levels of (*B*) TRAP5b activity and (*C*) CTX-I in culture media harvested at days 5, 7, and 9. TRAP5b activity was higher in *Ccr3*^*−/−*^ than *Ccr3*^*+/+*^ cultures between days 3 and 5, whereas the release of CTX-I was increased between days 5 to 7 and days 7 to 9 in *Ccr3*^*−/−*^ cultures compared with *Ccr3*^*+/+*^ cultures. Data represent mean values ± SD, n = 8 wells per genotype. ∗*p* < 0.05; ∗∗∗*p* < 0.001. CTX-1, C-terminal type I collagen; TRAP5b, tartrate-resistant acid phosphatase 5b.

To explore if CCR3 deficiency also affects osteoclast formation and morphology when cultured on a physiological substrate, and to investigate if the bone-resorbing capacity is affected, BMMs derived from *Ccr3*^*−/−*^ and *Ccr3*^*+/+*^ mice were cultured on bone slices. Similar to the results from BMM cultures on plastic, the *Ccr3*^*−/−*^ derived osteoclasts on bone slices were larger in size compared with the *Ccr3*^*+/+*^ derived cells, and the size difference visually increased over time (Fig. 2*A*). The amount of tartrate-resistant acid phosphatase 5b (TRAP5b) secreted into cell culture supernatants between days 3 and 5 was significantly higher in cultures of *Ccr3*^*−/−*^ osteoclasts compared with cultures of *Ccr3*^*+/+*^ osteoclasts (Fig. 2*B*). The release of C-terminal type I collagen (CTX-I, a marker of bone resorption) was significantly higher in cultures of *Ccr3*^*−/−*^ osteoclasts compared with cultures of *Ccr3*^*+/+*^ osteoclasts between days 5 to 7 and days 7 to 9 (Fig. 2*C*). Thus, osteoclasts lacking CCR3 show increased size and number of nuclei per osteoclast, higher TRAP5b release, and, when cultured on bone, an enhanced bone resorption capacity.

### CCR3 deficiency does not change expression of osteoclast-associated genes but coincides with upregulation of *Ccr2* and *Ccr5* expression

To investigate whether the different osteoclast morphology, seen in the absence of CCR3, is related to altered expression of established osteoclastogenic and osteoclastic genes, we performed quantitative PCR (qPCR) analyses in *Ccr3*^*−/−*^ and *Ccr3*^*+/+*^ BMM cultures supplemented with M-CSF or M-CSF and RANKL. RANKL upregulated all analyzed osteoclastogenic and osteoclastic genes downstream RANK in both genotypes (data not shown). However, no significant differences in mRNA levels of the pivotal osteoclastogenic transcription factors (Finkel–Biskis–Jinkins osteosarcoma oncogene [*c-Fos*] and nuclear factor–activated T cells c1 [*Nfatc1*]), the osteoclastogenic genes (receptor activator of nuclear factor–κB [*Rank*], Fc receptor γ subunit [*Fcer1g*], osteoclast-associated receptor [*Oscar*], alpha v beta 3 integrin [*Itgav*], DNAX adaptor protein 12 [*DAP12*]), and the osteoclastic genes (*Trap/Acp5*, cathepsin K [*Ctsk*], matrix metallopeptidase 9 [*Mmp9*], chloride channel 7 [*Clcn7*], calcitonin receptor [*Calcr*], dentrocyte expressed seven transmembrane protein [*Dcstamp*] or ATPase, H+ transporting, lysosomal V0 subunit D2 [*ATP6v0d2*]) were detected between the *Ccr3*^*−/−*^ and *Ccr3*^*+/+*^ cells (Table S2).

**Figure 3:**
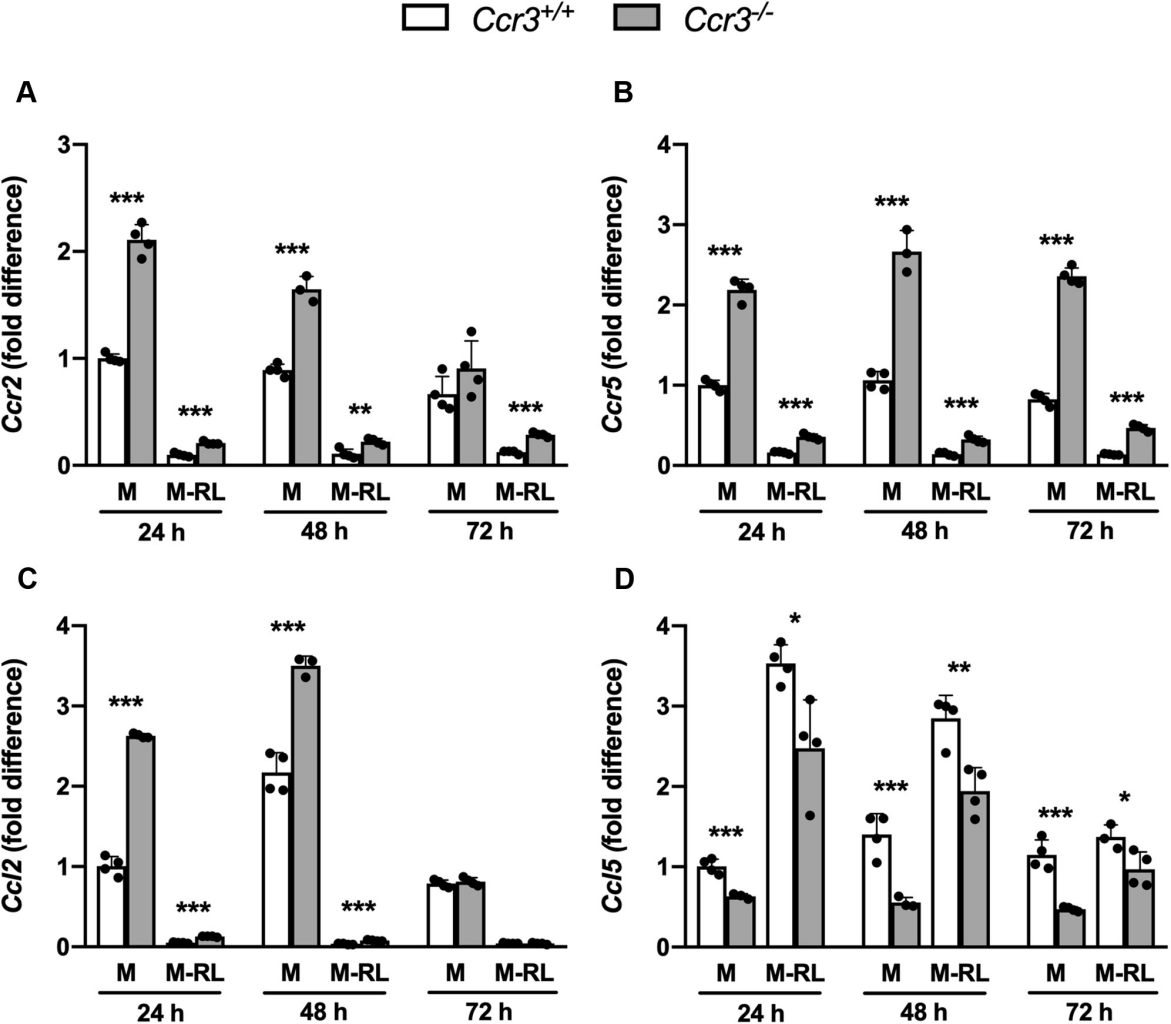
**Increased mRNA expression of *Ccr2* and *Ccr5* and the high-affinity ligand *Ccl2*, in *Ccr3***^***−/−***^**bone marrow macrophage cultures.** Bone marrow macrophages derived from *Ccr3*^*−/−*^ and *Ccr3*^*+/+*^ mice were cultured on plastic with macrophage colony-stimulating factor (M-CSF;M) alone or with M-CSF and receptor activator of nuclear factor kappa-B ligand (RANKL; M-RL). The cells were harvested, and the mRNA expression of *Ccr2*, *Ccr5*, *Ccl2*, and *Ccl5* was analyzed after 24, 48, and 72 h of incubation. *A*–*C*, the mRNA expression of *Ccr2*, *Ccr5*, and *Ccl2* was higher in *Ccr3*^*−/−*^ than *Ccr3*^*+/+*^ in M-CSF group, notably after 24 and 48 h and decreased in response to RANKL in both genotypes. *D*, contrary, the mRNA expression of *Ccl5* increased in response to RANKL in both genotypes, and the levels of *Ccl5* mRNA were even lower in *Ccr3*^*−/−*^ osteoclasts compared with *Ccr3*^*+/+*^ osteoclasts. Data represent mean values ± SD, n = 4 wells per genotype. ∗*p* < 0.05; ∗∗*p* < 0.01; and ∗∗∗*p* < 0.001.

Because the CCR3-related receptors CCR2 and CCR5 are both known to be important for osteoclast fusion and function, we also analyzed the mRNA expression levels of *Ccr2* and *Ccr5* and their high-affinity ligands *Ccl2* and *Ccl5*. In the BMM cultures supplemented with M-CSF, we observed significantly higher levels of *Ccr2*, *Ccl2*, and *Ccr5* mRNAs in *Ccr3*^*−/−*^ than in *Ccr3*^*+/+*^ cells, and this difference was apparent up to 48 h (*Ccr2* and *Ccl2*) and 72 h (*Ccr5*) after seeding (Fig. 3, *A*–*C*). Moreover, the *Ccl5* expression levels were lower in the *Ccr3*^*−/−*^ cells under these conditions (Fig. 3*D*). Although the addition of RANKL led to downregulation of the *Ccr2*, *Ccl2*, and *Ccr5* mRNAs in both *Ccr3*^*+/+*^ and *Ccr3*^*−/−*^ cells, the abundance of these mRNAs remained higher in the *Ccr3*^*−/−*^ cells (Fig. 3, *A*–*C*). RANKL caused upregulation of *Ccl5* in both *Ccr3*^*+/+*^ and *Ccr3*^*−/−*^ cells (Fig. 3*D*), although the expression levels were lower in the *Ccr3*^*−/−*^ cells. As expected, the expression of *Ccr3* was upregulated by the RANKL stimulation in the *Ccr3*^*+/+*^ cells (data not shown). Taken together, CCR3 deficiency does not change the expression of osteoclast-associated genes in response to RANKL but coincides with a higher expression of *Ccr2*, *Ccr5*, and *Ccl2* and lower expression of *Ccl5*.

### Addition of CCL5 inhibits the formation of giant hypernucleated osteoclasts

**Figure 4:**
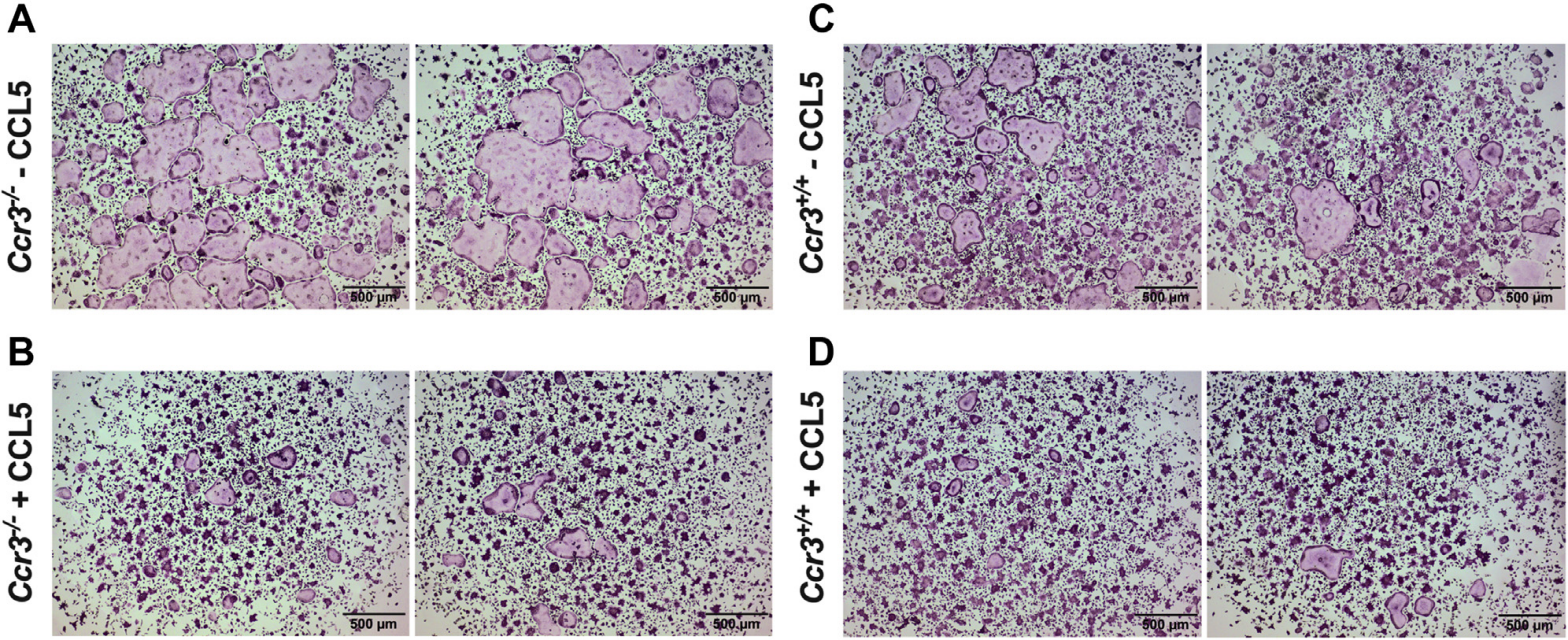
**Reduced formation of giant and hypernucleated *Ccr3***^***−/−***^**osteoclasts after addition of CCL5.** Bone marrow macrophages (BMMs) derived from *Ccr3*^*+/+*^ and *Ccr3*^*−/−*^ mice were cultured on plastic, supplemented with macrophage colony-stimulating factor and receptor activator of nuclear factor kappa-B ligand, in the presence and absence of CCL5 (1 μg/ml). *A*, in the absence of CCL5, *Ccr3*^*−/−*^ BMMs developed into large and hypernucleated osteoclasts after 72 h of culture, whereas (*B*) *Ccr3*^*−/−*^ BMMs cultured in presence of exogenous CCL5 showed reduced capacity to form oversized osteoclasts. However, the overall number of multinucleated osteoclasts formed was not reduced. Similarly, (*C*) *Ccr3*^*+/+*^ BMMs developed into multinucleated osteoclasts after 72 h of culture and (*D*) became smaller in size when cultured in presence of exogenous CCL5. Representative images are shown from one of two comparable experiments, n = 4 wells. CCL5, C–C motif chemokine ligand 5.

Because *Ccr3*^*−/−*^ preosteoclasts and osteoclasts show lower *Ccl5* and higher *Ccr5* mRNA abundance than the corresponding *Ccr3*^*+/+*^ cells, we investigated if addition of recombinant CCL5 could influence the osteoclast *in vitro* phenotype. BMMs derived from *Ccr3*^*+/+*^ and *Ccr3*^*−/−*^ mice were cultured for 72 h, supplemented with M-CSF and RANKL, in the presence and absence of CCL5. As in previous osteoclast cell culture experiments, *Ccr3*^*−/−*^ osteoclasts became very large in size (Fig. 4*A*). Addition of CCL5 clearly inhibited the formation of hypernucleated giant osteoclasts in *Ccr3*^*−/−*^ cultures (Fig. 4*B*) and also reduced the osteoclast size in *Ccr3*^*+/+*^ cultures (Fig. 4, *C*–*D*).

### CCR3 deficiency causes larger marrow space and lower cortical bone volume and thickness in mouse long bones

**Figure 5:**
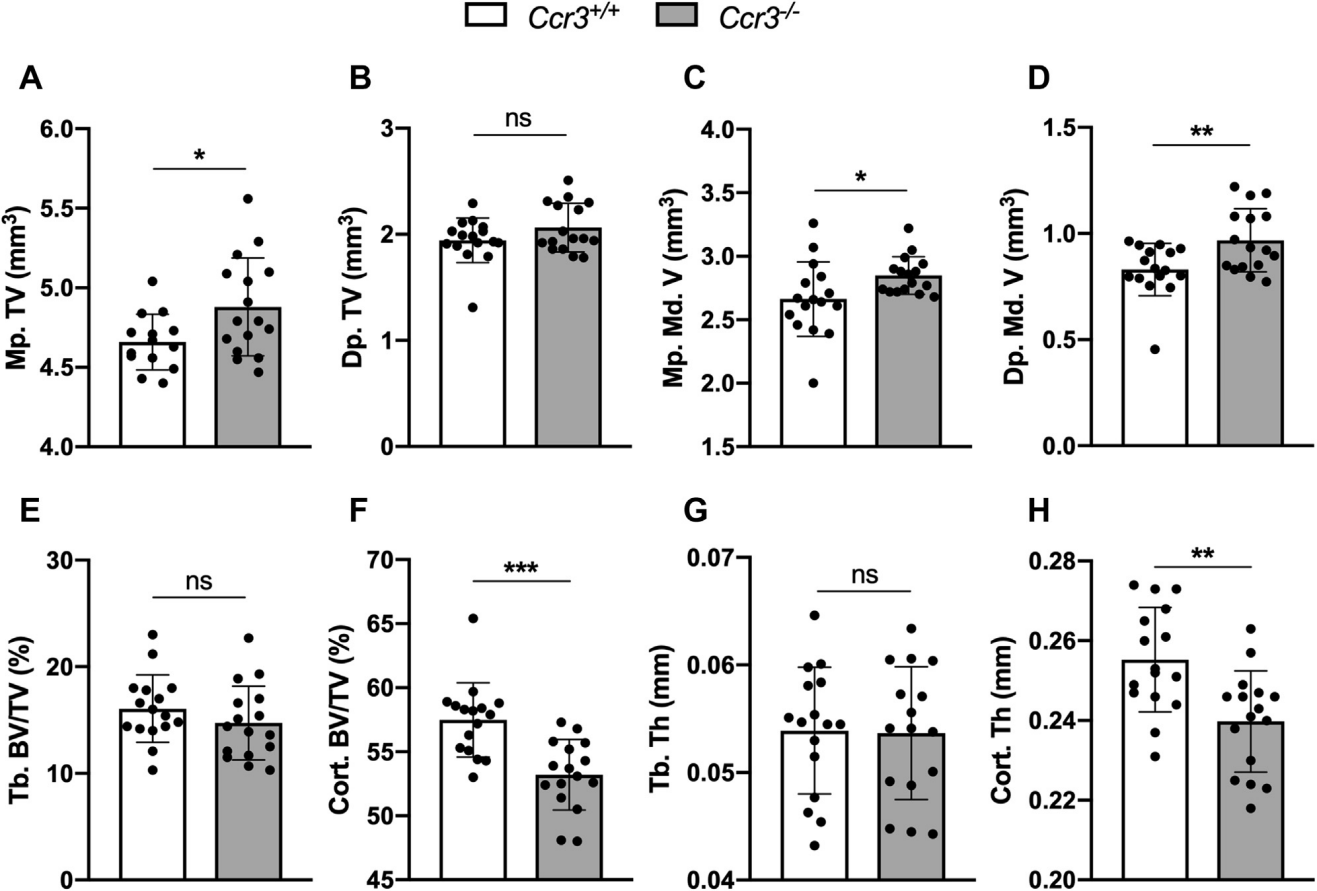
**Increased marrow space and reduced cortical bone volume and thickness in *Ccr3***^*−/−*^**long bones.** Microcomputed tomography analysis was performed on femurs to compare trabecular and cortical bone parameters. *A*–*B*, the total tissue volume (TV, mm^3^) measured at the metaphysis (Mp.) was significantly increased in *Ccr3*^*−/−*^ mice compared with *Ccr3*^*+/+*^ mice, whereas the total tissue volume measured at the diaphysis (Dp.) did not differ between the two genotypes. *C*–*D*, the marrow space, described as the medullary volume (Md. V, mm^3^), was significantly increased at both metaphysis and diaphysis in *Ccr3*^*−/−*^ mice. *E*–*F*, no difference in trabecular bone volume (Tb. BV/TV, %) could be seen between the genotypes, whereas the cortical bone volume (Cort. BV/TV, %) was significantly decreased in *Ccr3*^*−/−*^ mice compared with *Ccr3*^*+/+*^ mice. *G*–*H*, similarly, the trabecular thickness (Tb. Th, mm) was not altered in *Ccr3*^*−/−*^ mice, and the cortical thickness (Cort. Th, mm) was decreased compared with femurs from *Ccr3*^*+/+*^ mice. Data represent mean values ± SD, n = 16 per genotype. ∗*p* < 0.05; ∗∗*p* < 0.01; ∗∗∗*p* < 0.001. ns, not significant.

To determine if CCR3 deficiency affects bone parameters *in vivo*, microcomputed tomography (μCT) analyses were performed on dissected femurs of *Ccr3*^*+/+*^ and *Ccr3*^*−/−*^ mice. Trabecular and cortical bone parameters were measured at metaphysis and diaphysis, respectively. Even though the femur length did not differ between the two genotypes (average 16.29 mm in *Ccr3*^*+/+*^ and 16.09 mm in *Ccr3*^*−/−*^), μCT data analyses revealed that the total tissue volume, the volume inside the periosteum, was significantly higher at the metaphysis in *Ccr3*^*−/−*^ femurs (Fig. 5*A*), whereas it was equal for both genotypes at the diaphysis (Fig. 5*B*). The medullary volume, the volume between endosteum, was significantly higher in *Ccr3*^*−/−*^ mice at the metaphysis (Fig. 5*C*) and the diaphysis (Fig. 5*D*) compared with *Ccr3*^*+/+*^ mice. Further analyses of μCT data demonstrated that there was no difference in trabecular bone volume between the genotypes (Fig. 5*E*), and the cortical bone volume was significantly lower in *Ccr3*^*−/−*^ mice (Fig. 5*F*). Likewise, no difference in trabecular thickness could be observed in *Ccr3*^*−/−*^ compared with *Ccr3*^*+/*+^ mice, but the cortical thickness was significantly lower in *Ccr3*^*−/−*^ mice (Fig. 5, *G*–*H*, respectively). The trabecular number and pattern in *Ccr3*^*−/−*^ mice were not altered in comparison with *Ccr3*^*+/+*^ mice, although the trabecular separation was significantly higher in *Ccr3*^*−/−*^ mice (Fig. S3, *A*–*C*). This positively correlates to the larger marrow space and metaphyseal tissue volume.

### CCR3 deficiency causes increased mineralization rate of endocortical osteoid and higher trabecular and cortical bone formation rate in mouse long bones

**Figure 6:**
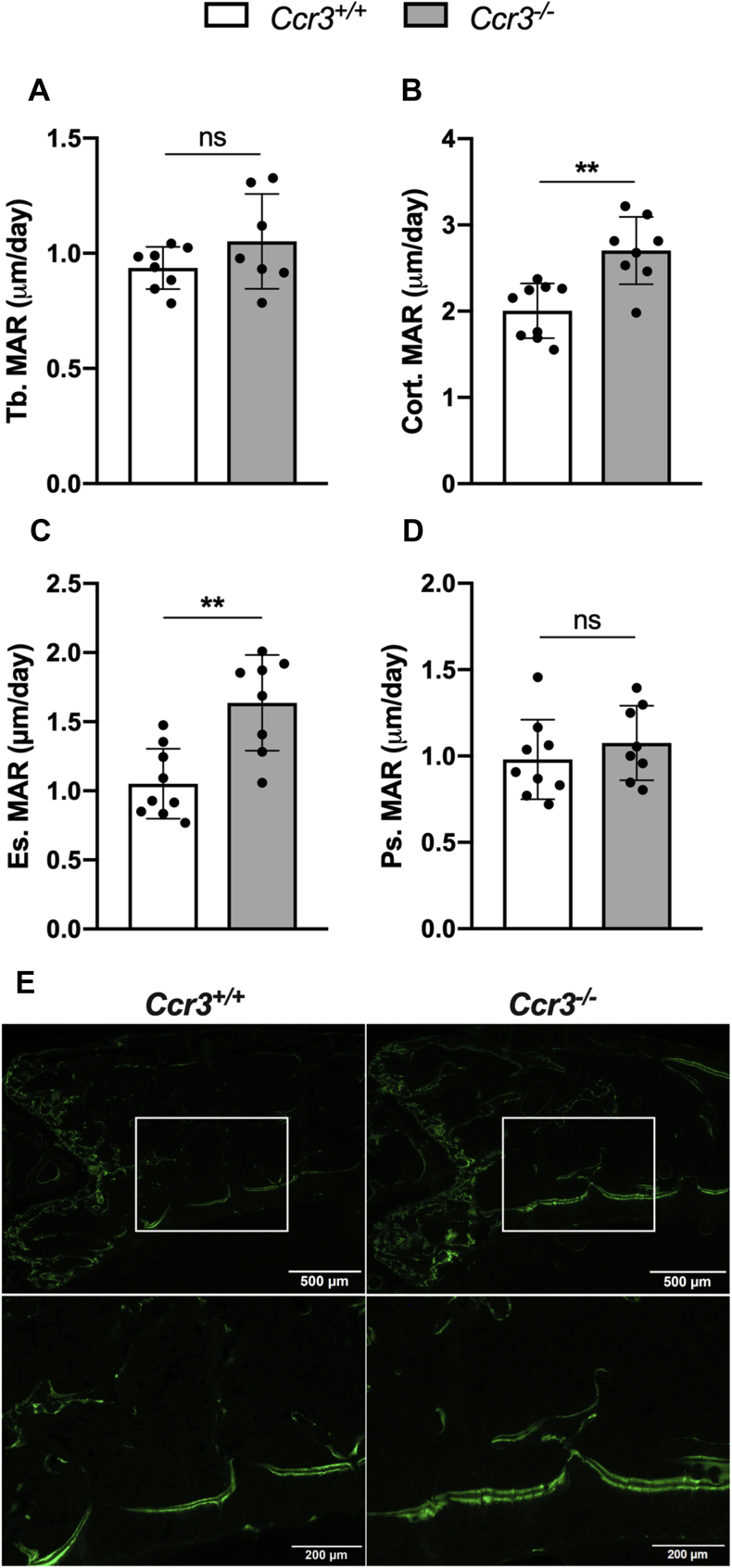
**Increased mineralization of endocortical osteoid in *Ccr3***^***−/−***^**long bones.** Quantification of mineral apposition rate (MAR) in sagittal bone sections from calcein-injected *Ccr3*^*+/+*^ and *Ccr3*^*−/−*^ mice showed (*A*–*D*) no differences in trabecular MAR (Tb. MAR, μm/day) between the genotypes, whereas the cortical MAR (Cort. MAR, μm/day) was increased in *Ccr3*^*−/−*^ mice compared with *Ccr3*^*+/+*^ mice. The augmented cortical MAR was foremost detected in the endosteal compartment (Es. MAR, μm/day), although the periosteal MAR (Ps. MAR, μm/day) was not significantly changed. *E*, representative images of fluorescent-labeled *Ccr3*^*+/+*^ and *Ccr3*^*−/−*^ bone sections, where an increased interlabel distance and thickness of the fluorescent bone labels could be seen on endosteal side of the cortical bone in the *Ccr3*^*−/−*^ section. Data represent mean values ± SD, n = 7 to 9 per genotype. ∗∗*p* < 0.01. ns, not significant.

**Figure 7:**
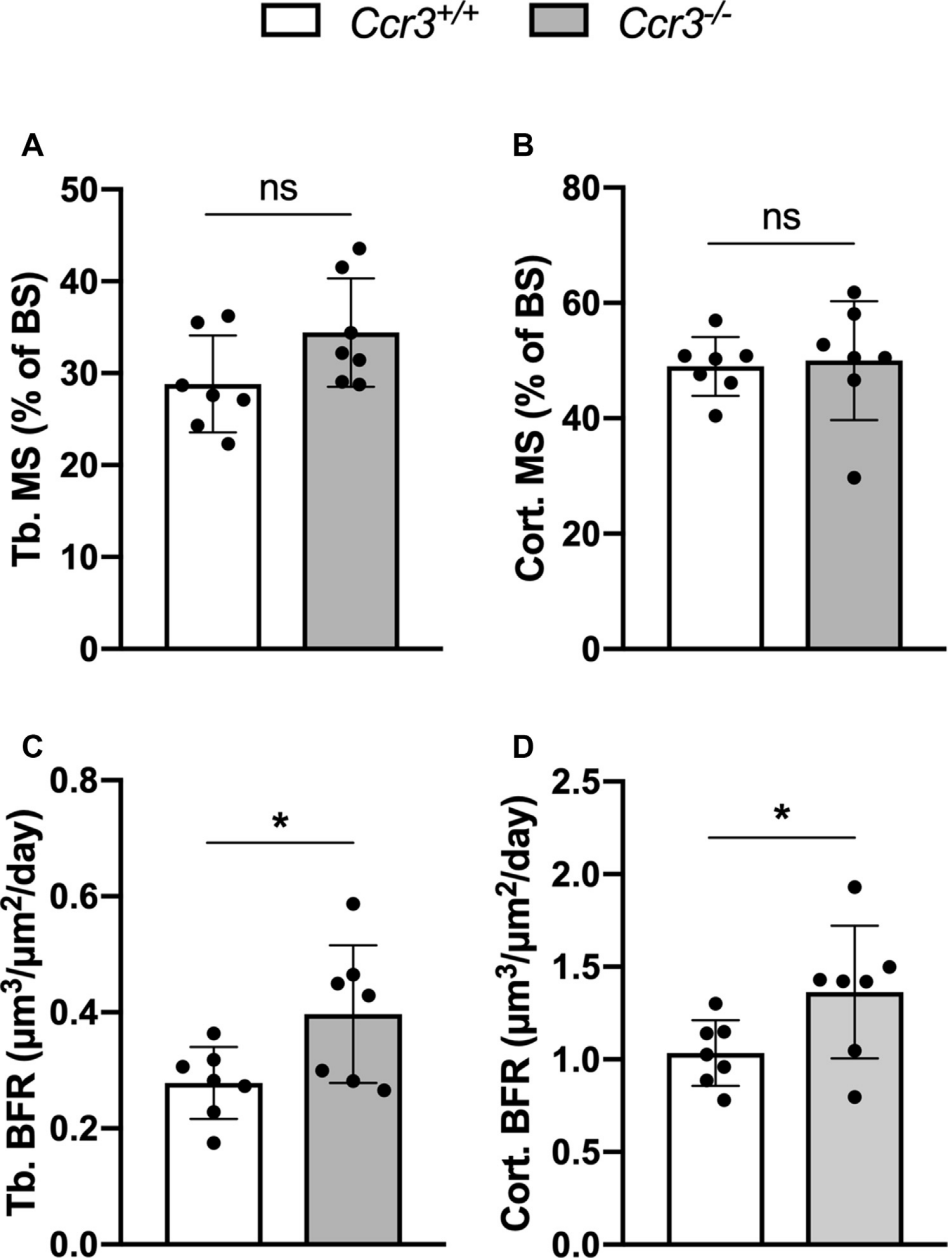
**Increased bone formation rate (BFR) in *Ccr3***^*−/−*^**long bones.** Quantification of (*A*–*B*) mineralizing surface (trabecular—Tb. MS; cortical—Cort. MS) and (*C*–*D*) BFR (trabecular—Tb. BFR; cortical—Cort. BFR) in sagittal bone sections from calcein-injected *Ccr3*^*+/+*^ and *Ccr3*^*−/−*^ mice showed significantly increased BFR in *Ccr3*^*−/−*^ mice compared with *Ccr3*^*+/+*^ mice. Data represent mean values ± SD, n = 7 per genotype. ∗*p* < 0.01. ns, not significant.

**Figure 8:**
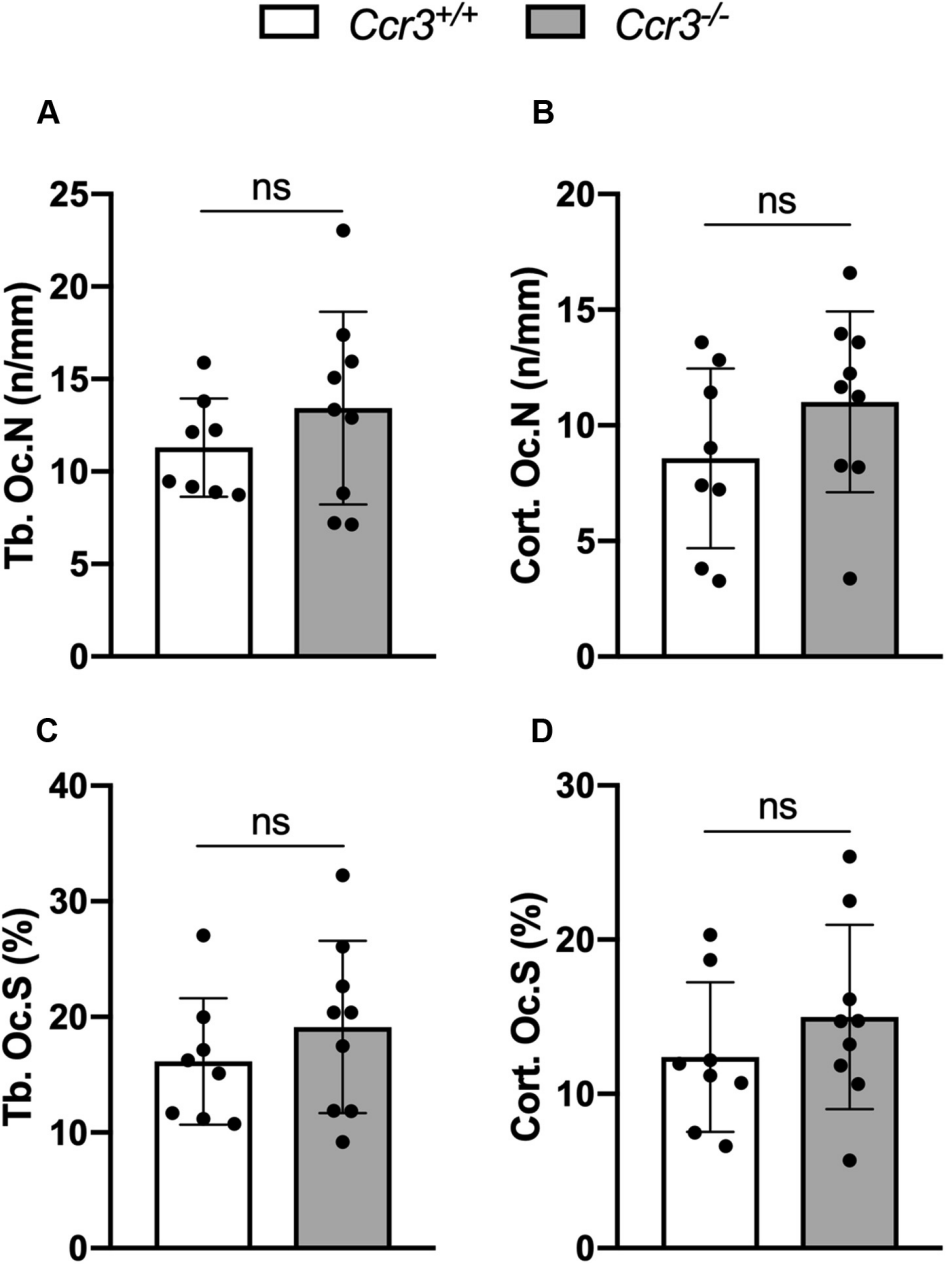
**No differences in osteoclast number and surface per millimeter of bone in *Ccr3***^*−/−*^**long bones.** Quantification of tartrate-resistant acid phosphatase (TRAP)^+^ (*A*–*B*) osteoclast number and (*C*–*D*) osteoclast surface per millimeter of bone (trabecular—Tb. Os.N: Tb. Os.S; cortical—Cort. Os.N; Cort. Os.S) in *Ccr3*^*+/+*^ and *Ccr3*^*−/−*^ bone sections showed no significant difference between the two genotypes. Data represent mean values ± SD, n = 8 to 9 per genotype. *ns*, not significant.

Dynamic histomorphometry was performed to evaluate the mineral apposition rate (MAR), mineralizing surface (MS), and bone formation rate (BFR) in nondecalcified bone sections from *Ccr3*^*+/+*^ and *Ccr3*^*−/−*^ femurs. The trabecular MAR was not significantly affected by the lack of CCR3 (Fig. 6*A*), whereas the cortical MAR was 35% higher in *Ccr3*^*−/−*^ mice (Fig. 6*B*). The augmented MAR was particularly found in the endosteal compartment with an increase of 56% in *Ccr3*^*−/−*^ mice compared with *Ccr3*^*+/+*^ mice (Fig. 6*C*). The periosteal MAR did not differ between the genotypes (Fig. 6*D*). Images of fluorescently labeled *Ccr3*^*+/+*^ and *Ccr3*^*−/−*^ distal femur bone sections show the larger interlabel distance and thicker fluorescent bone labels on the endosteal side of the cortical bone in the *Ccr3*^*−/−*^ section (Fig. 6*E*). No difference in trabecular or cortical MS could be seen between the genotypes, whereas the trabecular and cortical BFR was significantly increased by 43% and 32%, respectively, in *Ccr3*^*−/−*^ mice (Fig. 7, *A*–*D*). To further elucidate if the cortical bone phenotype in *Ccr3*^*−/−*^ mice is correlated to alterations in the number of osteoclasts or differences in osteoclast size *in vivo*, static histomorphometric analyses were performed on TRAP-stained bone sections from *Ccr3*^*−/−*^ and *Ccr3*^*+/+*^ mice. The number of osteoclasts in trabecular and cortical bones showed no significant difference between the genotypes (Fig. 8, *A*–*B*). In addition, no difference in osteoclast surface covering the underlying bone tissue could be seen (Fig. 8, *C*–*D*).

### Bone resorption and bone formation markers in serum are not affected by CCR3 deficiency

**Figure 9:**
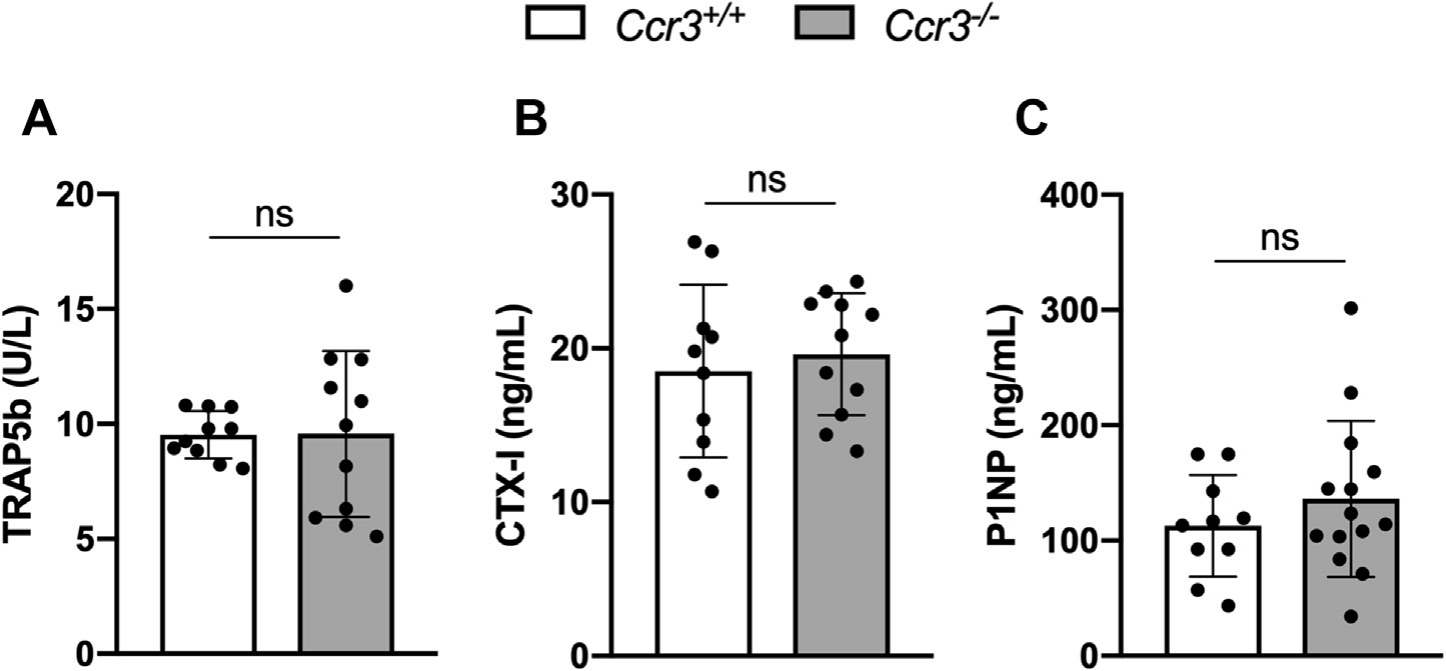
**No alterations in serum levels of bone resorption/formation markers in *Ccr3***^*−/−*^**mice.** Serum levels of (*A*) TRAP5b, (*B*) CTX-I, and (*C*) P1NP measured by ELISA showed no difference between the genotypes. Data represent mean values ± SD, n = 10 to 14 per genotype. CTX-1, C-terminal type I collagen; *ns*, not significant; P1NP, amino propeptide of type 1 collagen; TRAP5b, tartrate-resistant acid phosphatase 5b.

Because of the skeletal changes seen in the μCT and histomorphometry analyses, we analyzed bone turnover markers in blood serum from *Ccr3*^*−/−*^ and *Ccr3*^*+/+*^ mice. There were no significant differences, neither in the serum levels of TRAP5b, the bone resorption marker CTX-I, nor in the bone formation marker amino propeptide of type 1 collagen between genotypes (Fig. 9, *A*–*C*, respectively).

### CCR3 deficiency increases osteoblastic *Rankl* expression and the expression of osteoblast anabolic genes

**Figure 10:**
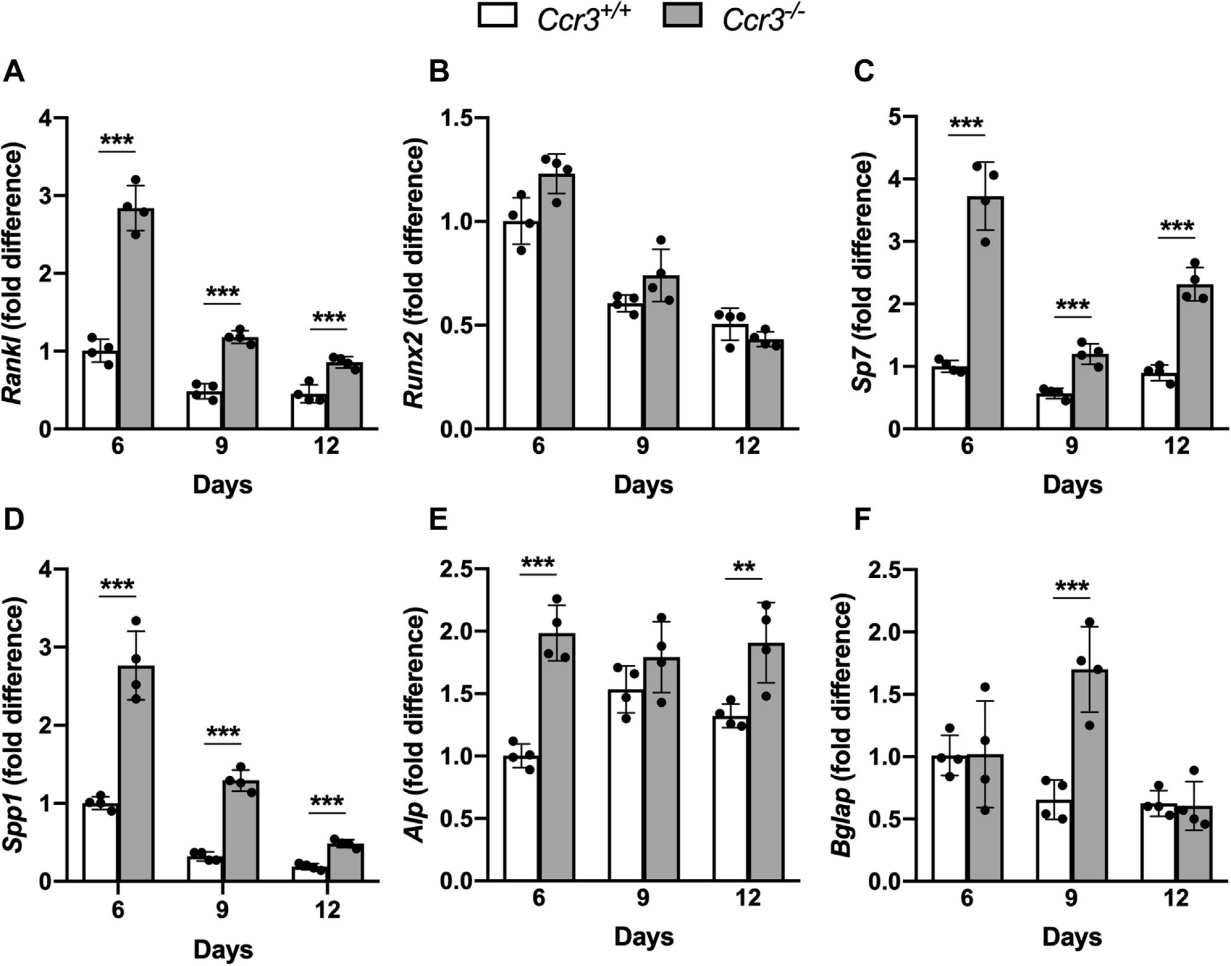
**Increased mRNA expression of *Rankl* and the osteoblast differentiation-associated genes *Sp7*, *Spp1*, *Alp*, and *Bglap* in *Ccr3***^***−/−***^**primary osteoblast cell cultures.** Primary osteoblasts isolated from *Ccr3*^*−/−*^ and *Ccr3*^*+/+*^ mice calvaria were cultured on plastic for 6 to 12 days before gene expression analysis. *A*–*F*, the mRNA expression of *Rankl*, *Sp7*, *Spp1*, *Alp*, and *Bglap* was significantly increased in *Ccr3*^*−/−*^ osteoblasts compared with *Ccr3*^*+/+*^ osteoblasts, whereas no difference could be seen in the mRNA expression of *Runx2* between the two genotypes. Data represent mean values ± SD, n = 4 wells per genotype. ∗∗*p* < 0.01; ∗∗∗*p* < 0.001.

To investigate if osteoblasts contribute to the disturbed bone remodeling in *Ccr3*^*−/−*^ mice, we isolated osteoblasts from *Ccr3*^*+/+*^ and *Ccr3*^*−/−*^ mice calvaria and cultured these primary osteoblasts for 6 to 12 days and performed gene expression analyses. The analyses showed that the osteoclast activator–related gene, *Rankl*, is upregulated in *Ccr3*^*−/−*^ osteoblasts (Fig. 10*A*). Although the expression of the early differentiation transcription factor runt-related transcription factor 2 (*Runx2*) was comparable in *Ccr3*^*+/+*^ and *Ccr3*^*−/−*^ osteoblasts (Fig. 10*B*), the level of *Sp7* mRNA, coding for the regulatory transcription factor osterix, was higher in the *Ccr3*^*−/−*^ osteoblasts after 6, 9, and 12 days of culture (Fig. 10*C*). Similarly, the level of *Spp1* mRNA, encoding the bone matrix protein osteopontin, was significantly higher in the *Ccr3*^*−/−*^ osteoblasts at all three time points (Fig. 10*D*). In addition, higher levels of the *Alp* and *Bglap* mRNAs (encoding the enzyme for mineral deposition alkaline phosphatase and the bone matrix protein osteocalcin, respectively) were detected in *Ccr3*^*−/−*^ osteoblasts after 6, 12, and 9 days of culture, respectively (Fig. 10, *E*–*F*).

## Discussion

In this article, we show that the absence of CCR3 receptors cause thinner cortical bone and an elevated mineral formation and BFRs in adult male mice. *In vitro* analyses reveal that CCR3 deletion is coupled to higher mRNA expression of *Ccl2*, *Ccr2*, and *Ccr5*, together with lower *Ccl5* mRNA expression, in preosteoclasts and osteoclasts. In addition, we show that the osteoclast size and activity are increased when CCR3 is missing. Moreover, primary mouse calvarial osteoblasts isolated from CCR3-deficient mice show increased mRNA expression of the osteoclast activator–related gene *Rankl*, as well as of genes encoding proteins associated with anchorage of osteoclasts and osteoblast differentiation.

We and others have shown that the *Ccr3* receptor mRNA expression is upregulated during RANKL-induced osteoclastogenesis (7, 19), but it is unknown what significance the receptor has for osteoclast formation. In this study, we used CCR3-deficient mice and show that lack of CCR3 receptors causes formation of giant hypernucleated osteoclasts that display enhanced bone resorption *in vitro*. *In vivo* skeletal analyses show that the number of osteoclasts were equivalent in both CCR3-deficient and CCR3-proficient mice long bones, which corresponds to our observations from *in vitro* analyses. *In vivo*, however, we could not detect any difference in size of osteoclasts. One reason behind these discrepancies could be that two different units of measurements were compared, *i.e.*, an area *in vitro* with a length *in vivo*, which generally does not reflect the whole three-dimensional size of the cells. The size of osteoclasts *in vitro* is estimated as an area measured in several wells of cell cultures, whereas *in vivo* as a length (millimeter) of osteoclasts estimated on one single section per mouse. Unfortunately, it is with current method impossible to estimate the size of an osteoclast and the number of nuclei per osteoclast on bone sections. *In vitro*, CCR3-deficient osteoclasts seeded on bone substrate show large osteoclasts with many nuclei that very efficiently resorb bone. This might be coupled to enhanced recruitment and fusion of preosteoclasts and/or increased osteoclast adherence, motility, and/or resorptive capacity. If this corresponds to the *in vivo* situation with osteoclasts on bone surfaces (BSs), the thinner cortical bone detected in the CCR3-deficient mice could be due to increased osteoclast activity when CCR3 is missing.

Chemokine signaling commonly stimulates osteoclastogenesis and osteoclast activity in an indirect manner through upregulation of RANK and subsequent downstream signaling proteins (13, 14, 20). Most surprisingly, none of the analyzed genes associated with osteoclast differentiation or fusion were expressed differently in CCR3-deficient osteoclast cultures. Our finding of higher *Ccl2*, *Ccr2*, and *Ccr5* gene expression could indirectly contribute to the increased fusion of osteoclasts, creating giant hypernucleated osteoclasts, and the heightened bone resorptive activity seen in CCR3-deficient osteoclast cultures. CCR5 is known to stimulate cellular locomotion and bone resorption activity of osteoclasts, which is associated with the arrangement of podosomes and adhesion complex molecules including Pyk2 (15). Thus, the increased *in vitro* bone resorption in the CCR3-deficient osteoclast cultures on bone slices may be explained by increased osteoclast locomotion as the osteoclast function genes are not upregulated. Consistent with this notion, increased osteoclastogenesis is previously described in mice lacking CCL5 (18), indicating that CCL5 inhibits osteoclast formation. Besides higher *Ccr5* mRNA expression in our CCR3-deficient BMM cultures, we also detected lower *Ccl5* mRNA levels. Furthermore, addition of exogenous CCL5 to CCR3-deficient BMM cultures completely abolished the formation of giant hypernucleated osteoclasts, implying that the increased *Ccr5* expression and decreased *Ccl5* expression drive formation of giant osteoclasts when CCR3 is absent. As for CCR2 and CCL2, Khan *et al.* (21) could show that osteoclast formation was impaired in mice lacking a functional copy of either gene. For instance, a lower number of osteoclasts, accompanied by a reduction in cell size and a lower number of nuclei per cell, were evident in CCR2- and CCL2-deficient mice. Moreover, addition of exogenous CCL2 increased the number of osteoclasts and number of nuclei seen in CCL2-proficient cell cultures and restored the number and size of osteoclasts in CCL2-deficient cultures. Interestingly, the changed osteoclast morphology seen in CCR2-deficient mice was accompanied by higher levels of CCR3, suggesting that CCR3 contributes to a reduction of osteoclast size and nucleation (21). Therefore, our observation of increased *Ccl2* and *Ccr2* expression in CCR3-deficient BMM cultures may explain the formation of oversized osteoclasts. However, whether the pervading formation of giant osteoclasts and increased osteoclast activity seen in the absence of CCR3 occur indirectly because of upregulation of CCL2 and CCR2/CCR5 receptors, and downregulation of CCL5, needs to be further investigated.

The thin cortical femur bones found in the CCR3-deficient mice could be due to increased bone resorption, decreased bone formation, or a combination of both. Interestingly, our dynamic histomorphometric analyses demonstrate an increased cortical mineralization rate of osteoid, mainly in the endosteal compartment, and that the BFR was increased in both cortical and trabecular bones in CCR3-deficient mice. This may be a compensatory mechanism to meet a high bone resorption activity or a sign of accelerated bone turnover, which requires increased osteoblast recruitment and activity.

Yano *et al.* (17) have shown that osteoblasts express CCR3 receptors, but the role of CCR3 signaling in osteoblasts is hitherto unexplored. We isolated and cultured mouse calvarial osteoblast from CCR3-proficient and CCR3-deficient mice and found that, during the whole culture period, the deletion caused higher mRNA expression of *Rankl*, the gene encoding the RANKL protein with a central role in osteoclastogenesis and activation of osteoclasts (1). Osteoblastic RANKL expression *in vivo* could contribute to increased preosteoclast recruitment and/or increased osteoclast activity. In this context, it is interesting that we found an increased expression of *Spp1* (osteopontin) mRNA in the CCR3-deficient osteoblasts. Osteopontin is important for, *e.g.*, anchorage of osteoclasts (22) and could, if osteopontin is enriched in the CCR3-deficient mouse skeleton, promote bone resorption. Interestingly, *Sp7* (osterix), a transcription factor crucial for driving osteoblast differentiation, was enhanced in CCR3-deficient osteoblast, along with *Spp1* (osteopontin), *Alp* (alkaline phosphatase), and *Bglap* (osteocalcin). Because the last two genes encode proteins that are coupled to mineralization of bone (23, 24), the CCR3-deficient osteoblasts show an increased anabolic capacity, which could contribute to the increased mineralization seen in our dynamic histomorphometric analyses of osteoid mineralization rate and BFR in CCR3-deficient mice. Clearly, the osteoblastic gene expression is changed when CCR3 is missing, not only in a way that could contribute to increased osteoclast recruitment, adherence, and activity but also in a bone anabolic direction.

In 2019, Mohan *et al.* (25) demonstrated that young female mice lacking CCR3 have increased trabecular bone mass because of a greater number and thickness of trabeculae compared with wildtype littermates. This is in contrast to our findings and could be explained by the differences in gender and age of the mice used in our respective studies. Moreover, our observation of an overall increased metaphyseal tissue and medullary volume indicates that the CCR3-deficient mice may have an altered femoral shape because of a changed postnatal skeletal development and growth. Thus, as our study is limited to one single model at one time point, further analyses are needed to investigate if CCR3 deficiency affects bone modeling in young male mice.

Although our skeletal analyses of the CCR3-deficient mice show an altered bone remodeling, our analyses of serum bone metabolic markers do not reveal any significant differences compared with the levels in CCR3-proficient mice. One explanation could be that the cortical bone, where we see the most distinct differences, in general is very slowly remodeled. This makes it difficult to track serum level differences when measuring at one single time point. Another explanation could be that the adult bone phenotype in CCR3-deficient mice is mainly mirroring early modeling effects. Notably though, the TRAP5b serum levels vary greatly in CCR3-deficient mice compared with CCR3-proficient mice. This may reflect the large variation in osteoclast size seen *in vivo*, corresponding to our *in vitro* observation with no increase in osteoclast numbers in CCR3-deficient cultures, but an increase in osteoclast size and TRAP5b levels compared with CCR3-proficient mice.

The fact that the CCR3 expression is upregulated in circulating monocytes of osteoporotic individuals with low bone mineral density (26) and in peripheral blood leukocytes in patients with RA (27) suggests that the receptor is involved in the disturbed bone remodeling process. Further studies are needed to rule out if the increased CCR3 expression is a defense mechanism prohibiting recruitment of osteoclast progenitor cells. We and others have shown that the CCR3 ligand CCL11, which is not synthesized by monocytes or osteoclasts, is associated with inflammatory bone loss conditions (6, 8) and that CCL11 stimulates preosteoclast migration and bone resorption *in vitro*. However, it is not clear by which receptor these effects are mediated. Studying chemokine receptor signaling using antibodies is difficult as the crossreactivity is high. To address the role of different chemokine ligands, and the potential functional interplay between chemokine receptors, such as CCR2, CCR3, and CCR5, conditionally genetic deficient cell and animal models are needed.

Taken together, the present study further emphasizes the relevance of studying the role of a hitherto virtually unrecognized player in bone research, CCR3, in bone cell differentiation and activity.

## Experimental procedures

### Mice

Balb/C.129S4-*Ccr3*^tm1cge^/J wildtype and heterozygous mice were obtained from The Jackson Laboratory (Bar Harbor, ME, USA). Breeding was performed to generate *Ccr3*^*−/−*^ mice and wildtype/heterozygous littermates. Animals were housed at 12:12 h dark/light cycle in a temperature-/humidity-controlled (22 °C/50%) room and *ad libitum* feeding standard chow (Special Diet Service #801730). Only male mice were used. Animal care and experiments were approved and conducted in accordance with regulations of the Local Animal Ethical Committee at Umeå University.

### BMMs

Femurs and tibiae from 4- to 8-week-old male mice were dissected and cleaned from adhering tissues. The marrow cavity was flushed, and M-CSF–induced BMMs were prepared as previously described (28). Briefly, after lysis of erythrocytes, cells were cultured in minimum essential medium Eagle, alpha modification (α-MEM) supplemented with 10% fetal bovine serum, L-glutamine (Life Technologies Ltd, Europe BV), and antibiotics (streptomycin, penicillin, and gentamycin) (Gibco, Grand Island, NY, USA) (hereafter referred to as complete α-MEM) and M-CSF (30 ng/ml) (R&D Systems/Biotechne, Abingdon, UK) in Corning nontreated culture dishes (Corning Inc, Corning, NY, USA) to which BMMs adhere but not stromal cells or lymphoid cells. After 3 days of incubation, nonadherent cells were discarded, and the adherent BMMs were harvested and either used for immediate RNA isolation and qPCR analyses or seeded for continued culturing as described later.

### Osteoclast formation in cultures on plastic dishes

BMMs as prepared previously were seeded in droplets of 5 μl (5 × 10^3^ cells) in 96-multiwell plates (Nunc, Roskilde, Denmark). The cells were left to adhere for 10 min whereafter 200 μl of complete α-MEM supplemented with M-CSF (30 ng/ml) and with or without RANKL (4 ng/ml) (R&D Systems/Biotechne) and CCL5 (1 μg/ml) (R&D Systems/Biotechne) was added. The cells were incubated at 37 °C in 5% CO_2_ for various times (designated in Results section), whereafter the culture media were harvested and stored in aliquots at −20 °C until analysis of the active isoform 5b of TRAP5b (Immunodiagnostic systems [IDS], Copenhagen, Denmark). Adherent cells were fixed at the bottom of the 96-well plate and stained for TRAP using the leukocyte acid phosphatase kit (Sigma–Aldrich, St Louis, MO, USA), following the manufacturer's instruction. TRAP-positive cells with three or more nuclei were considered osteoclasts. Images were acquired using an Olympus BX41 light microscope. For gene expression analyses, BMMs were seeded in droplets of 40 μl (4 × 10^4^ cells) in 12-multiwell plates (Nunc) and cultured as mentioned previously.

### Osteoclast formation in cultures on bone slices

BMMs were also seeded on bovine cortical bone slices (IDS) placed at the bottom of 96-well plates (Nunc) (5 × 10^3^ cells/well) in 200 μl of complete α-MEM, supplemented with M-CSF (30 ng/ml) and with or without RANKL (4 ng/ml) and incubated at 37 °C in 5% CO_2_ for 9 days. Culture media were changed at days 3, 5, and 7. The media from day 5, day 7, and at study discontinuation at day 9 were harvested and stored in aliquots at −20 °C until TRAP5b and CTX-I analysis (IDS).

### Primary osteoblast cell culture

Preosteoblast cells were isolated through enzymatic digestion of five calvaria from each genotype, using Worthington's type 2 collagenase (Worthington Biochemical Corporation, Lakewood, NJ, USA). Equivalent number of cells from both genotypes were seeded in separate 75 cm^2^ cell culture flasks and cultured in complete α-MEM in 37 °C and 5% CO_2_. Media were changed after 1 and 3 days of culture. After 4 days of culture, the cells were washed with EDTA and detached with 0.25% trypsin–EDTA. Following centrifugation and resuspension in complete α-MEM, the cell suspension was diluted to 40,000 cells/ml and seeded in 24-well plates at a density of 20,000 cells per well. The next day media were changed—day 0. Cells were cultured in 500 μl of complete α-MEM. Half of the media were changed twice a week, and all media were changed once a week to compensate for evaporation.

### RNA isolation and first-strand complementary DNA synthesis

Total RNA was extracted from cells using the RNAqueous Micro Kit (Ambion, Austin, TX, USA) and subsequently treated with DNase. Single-stranded complementary DNA was synthesized from 0.2 μg of total RNA using the High-Capacity complementary DNA Reverse Transcription Kit (Applied Biosystems, Foster City, CA, USA), as per the manufacturer's instructions.

### Real-time qPCR

Real-time qPCR analyses were performed in a QuantStudio 6 Flex real-time PCR system (Applied Biosystems by Life Technologies, Carlsbad, CA, USA) using TaqMan Universal Master Mix (Applied Biosystems) and predesigned TaqMan gene expression assays (Table S4).

Samples were run in duplicates, and the data were normalized to mRNA levels of the endogenous control gene, β2-microglobulin (*B2m*). The 2^−ΔΔCt^ formula was used to analyze the relative differences in gene expression (29).

### Microcomputed tomography

High-resolution μCT analysis was performed on femurs of 14- to 16-week-old male mice using Skyscan 1275 μCT (Bruker, Kontich, Belgium). Femurs were imaged with an X-ray tube voltage of 40 kV, a current of 250 μA, and with a 1 mm aluminum filter. The voxel size of 8 μm was used. NRecon software (version 1.7.3.1) was employed for image reconstruction. Region/volume of interest selection, segmentation of binary image, and morphometric analysis were all performed using SkyScan CT-Analyser (CTAn) software. Volume of interest for trabecular bone was selected at the distal femur within a conforming volume commencing at the growth plate and extending a further longitudinal distance of 0.48 mm (60 image slices) in the proximal direction and for cortical bone—4 mm (500 image slices). A total of 1.6 mm (200 image slices) and 1.2 mm (150 image slices) were analyzed for trabecular and cortical bone, respectively.

### Serum biomarkers

Blood serum was collected from 16-week-old mice. As a marker of bone resorption, serum levels of CTX-I were assessed using an ELISA RatLaps kit (IDS). To validate osteoclast activity, a determination of TRAP5b activity was performed using mouse TRAP5b ELISA kit (IDS). Serum levels of amino propeptide of type 1 collagen were analyzed as a marker of bone formation (IDS).

### Histomorphometry

About 18- to 19-week-old *Ccr3*^*+/+*^ and *Ccr3*^*−/−*^ mice were injected with fluorescent compound calcein (30 mg/kg) (Sigma–Aldrich) 10 days and 3 days prior to tissue collection. Dissected femurs from both genotypes were fixed in 4% phosphate-buffered paraformaldehyde (HistoLab Products AB, Askim/Gothenburg, Sweden) and embedded in methyl methacrylate (Pharmatest Services Ltd, Turku). Sagittal tissue sections (thickness of 4 and 8 μm) of distal femurs were used for quantification of dynamic bone histomorphometric parameters after fluorescent microscopy (Olympus BX41; Olympus CellSens Standard). The amount of trabecular and cortical MS (Tb. MS; Cort. MS, %) was estimated as the coverage of BS by fluorescent double-labeled surface and single-labeled surface according to the equation MS = (double-labeled surface + [single-labeled surface/2] × 100)/BS. Trabecular and cortical MAR (Tb. MAR; Cort. MAR, μm/day), including the periosteal and endosteal surfaces separately (Es. MAR; Ps. MAR, μm/day), was estimated by the interlabel distance of the two consecutive calcein labels divided by the time interval between the injections of labels. Corresponding BFR (μm^3^/μm^2^/day) was calculated as BFR = (MS/BS) × MAR. Adjacent sagittal sections were stained for mineralized tissue with von Kossa according to histochemical standard procedure, and the trabecular and cortical BS were analyzed by using a light microscope (Olympus BX41; Olympus CellSens Standard). Trabecular and cortical osteoclast number (Tb. Oc.N; Cort. Oc.N) and surface (Tb. Os.S; Cort. Oc.S) per millimeter of bone (expressed as n/mm and %) were quantified in tissue sections of paraffin-embedded femurs from *Ccr3*^*+/+*^ and *Ccr3*^*−/−*^ mice stained for TRAP positivity with leucocyte acid phosphatase kit (Sigma–Aldrich) according to manufacturer's instructions. In all samples analyzed for histomorphometric parameters, the region of interest was set as the area inferior of the epiphyseal growth plate seen in 20× magnification.

### Statistical analyses

The statistical analyses were performed using Student's *t* test, and all experiments were performed at least twice with comparable results. In the gene expression analyses, the ΔCt values were used (determined as the Ct value for the gene of interest minus the Ct value for the reference gene). All data are presented as means ± SD. Significance levels were set to *p* < 0.05 (∗), 0.01 (∗∗), and 0.001 (∗∗∗).

## Data availability

All relevant data are included in the article and the supporting information files. Original data are available on reasonable request.

## Conflict of interest

The authors declare that they have no conflicts of interest with the contents of this article.
