# Supplementary material for: CCR3 deficiency is associated with increased osteoclast activity and reduced cortical bone volume in adult male mice
Source: J Biol Chem. 2020 Dec 17;296:100177. doi: 10.1074/jbc.RA120.015571 (PMC7948475; doi:10.1074/jbc.RA120.015571)
Supplement: Supplementary Figures and Tables [file mmc1.pdf]

Supplement 1.

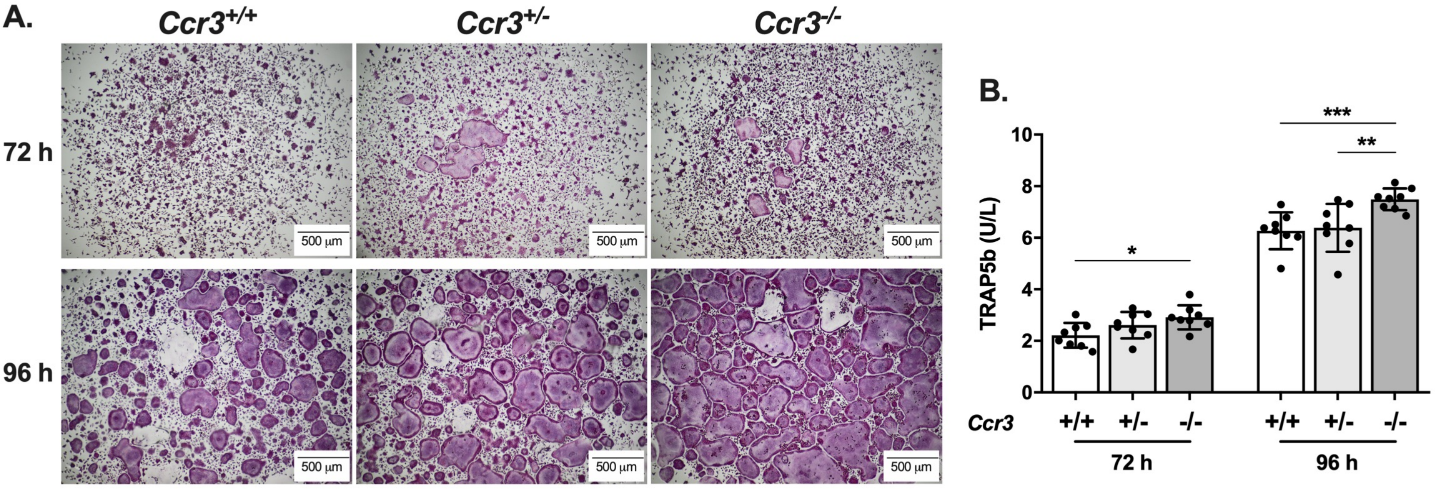

Supplement 1. Increased osteoclast size and TRAP5b-positivity in *Ccr3*$^{-/-}$ and *Ccr3*$^{+/-}$ BMM-cultures. BMMs derived from *Ccr3*$^{-/-}$*, Ccr3*$^{+/-}$ and *Ccr3*$^{+/+}$ mice were cultured on plastic with M-CSF and RANKL. **(A)** *Ccr3*$^{+/-}$ BMMs developed into large, oversized osteoclasts after 72 h and 96 h of incubation, although not as clear as *Ccr3*$^{-/-}$ BMMs. After 96 h of culture, a number of osteoclasts have detached and remnents of cell membranes are seen in the white areas. Representative images are shown. **(B)** Levels of TRAP5b in cell culture supernatants were significantly higher in *Ccr3*$^{-/-}$ than *Ccr3*$^{+/+}$ cultures after 72 h and higher than both *Ccr3*$^{+/-}$ and *Ccr3*$^{+/+}$ after 96 h of incubation. Data represent mean values ± SD, n=8 wells per genotype. *$p < 0.05$; **$p < 0.01$; ***$p < 0.001$.

Supplement 2.

Relative difference in the mRNA expression of osteoclast associated genes

			M-CSF		M-CSF + RANKL	
	**Gene**	**Time (h)**	**CCR3+/+**	**CCR3-/-**	**CCR3+/+**	**CCR3-/-**
Osteoclastogenic transcription factors	*C-fos*	24	1.02 ± 0.25	0.87 ± 0.09	4.96 ± 0.24	4.13 ± 0.54
		48	2.19 ± 0.43	0.78 ± 0.21	11.0 ± 0.83	6.84 ± 0.66
		72	0.14 ± 0.01	0.14 ± 0.01	2.66 ± 0.59	2.24 ± 0.06
	Nfatc1	24	1.02 ± 0.27	0.99 ± 0.17	9.24 ± 0.48	6.78 ± 0.57
		48	1.09 ± 0.17	1.20 ± 0.30	24.0 ± 0.79	19.3 ± 3.40
		72	0.92 ± 0.07	0.98 ± 0.19	16.4 ± 2.87	13.2 ± 0.57
Osteoclastogenic marker genes	*DAP12*	24	1.00 ± 0.11	1.18 ± 0.09	0.44 ± 0.02	0.43 ± 0.04
		48	0.96 ± 0.07	1.06 ± 0.19	0.77 ± 0.07	0.62 ± 0.07
		72	0.83 ± 0.05	0.84 ± 0.01	1.25 ± 0.23	1.06 ± 0.03
	Fcer1g	24	1.01 ± 0.16	0.97 ± 0.10	0.31 ± 0.01	0.31 ± 0.03
		48	0.92 ± 0.07	1.04 ± 0.10	0.37 ± 0.02	0.32 ± 0.02
		72	0.70 ± 0.03	0.74 ± 0.05	0.45 ± 0.02	0.36 ± 0.02
	Itgav	24	1.01 ± 0.21	0.95 ± 0.14	1.95 ± 0.21	1.41 ± 0.19
		48	0.79 ± 0.16	0.78 ± 0.12	6.74 ± 0.43	5.62 ± 0.98
		72	0.54 ± 0.02	0.54 ± 0.04	6.63 ± 1.35	5.73 ± 0.21
	Oscar	24	nd	nd	nd	nd
		48	nd	nd	144 ± 12.6	117 ± 27.9
		72	nd	nd	604 ± 103	536 ± 101
	Rank	24	1.02 ± 0.24	0.91 ± 0.14	0.74 ± 0.05	0.58 ± 0.09
		48	0.57 ± 0.10	0.60 ± 0.12	1.70 ± 0.09	1.45 ± 0.20
		72	0.31 ± 0.02	0.36 ± 0.03	1.68 ± 0.27	1.38 ± 0.05
Osteoclastic genes	*Acp5/Trap*	24	1.02 ± 0.26	0.81 ± 0.12	27.5 ± 3.18	19.6 ± 1.53
		48	0.41 ± 0.06	0.53 ± 0.11	344 ± 24.0	308 ± 72.5
		72	0.29 ± 0.02	0.37 ± 0.05	854 ± 105	809 ± 45.0
	Atp6v0d2	24	1.02 ± 0.25	1.35 ± 0.22	10.8 ± 0.73	7.85 ± 0.82
		48	0.70 ± 0.13	1.16 ± 0.18	118 ± 10.5	99.4 ± 11.1
		72	0.56 ± 0.10	0.82 ± 0.07	168 ± 35.5	135. ± 1.68
	Calcr	24	nd	nd	nd	nd
		48	nd	nd	62.5 ± 8.06	47.1 ± 12.8
		72	nd	nd	837 ± 106	554 ± 32.8
	Clcn7	24	1.00 ± 0.12	1.11 ± 0.17	1.01 ± 0.08	0.87 ± 0.11
		48	0.95 ± 0.13	1.25 ± 0.29	8.14 ± 1.04	7.75 ± 0.93
		72	0.92 ± 0.06	1.00 ± 0.04	20.4 ± 3.33	14.8 ± 1.28
	Ctsk	24	1.08 ± 0.53	0.43 ± 0.10	36.9 ± 5.66	22.9 ± 3.26
		48	0.27 ± 0.10	0.30 ± 0.07	1072 ± 147	886 ± 188
		72	0.12 ± 0.01	0.18 ± 0.11	2711 ± 503	2198 ± 144
	Dcstamp	24	1.02 ± 0.24	0.48 ± 0.10	44.3 ± 1.28	28.8 ± 2.71
		48	0.14 ± 0.10	0.10 ± 0.04	356 ± 28.4	292 ± 22.6
		72	0.07 ± 0.02	0.07 ± 0.05	538 ± 33.6	417 ± 28.5
	Mmp9	24	1.19 ± 0.80	0.36 ± 0.20	95.6 ± 13.1	60.9 ± 9.06
		48	0.15 ± 0.04	0.12 ± 0.01	1090 ± 73.4	789 ± 84.3
		72	0.06 ± 0.01	0.05 ± 0.02	2332 ± 187	1966 ± 143

Supplement 2. mRNA expression of established osteoclastogenic and osteoclastic genes in *Ccr3*$^{-/-}$ and *Ccr3*$^{+/+}$ BMM cultures. BMMs derived from *Ccr3*$^{-/-}$ and *Ccr3*$^{+/+}$ mice were cultured in plastic wells with M-CSF alone or M-CSF and RANKL. The cells were harvested after 24 h, 48 h or 72 h of incubation and the mRNA levels were determined using qPCR with predesigned TaqMan gene expression assays as listed in Suppl. 4. Data are presented as fold difference (mean ± SD) relative to *Ccr3*$^{+/+}$ BMMs incubated with M-CSF for 24 h. n=4 wells per data point, n.d.=not detectable.

Supplement 3.

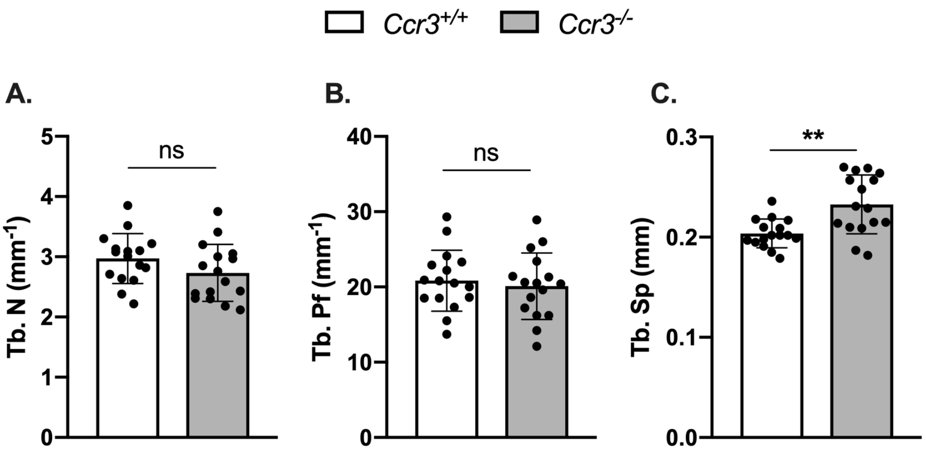

Supplement 3. Higher trabecular separation in *Ccr3*$^{-/-}$ long bones. µCT analyses of *Ccr3*$^{+/+}$ and *Ccr3*$^{-/-}$ femurs to compare **(A)** trabecular number (Tb. N), **(B)** trabecular pattern factor (Tb. Pf) and **(C)** trabecular separation (Tb. Sp), showed no alterations in number and pattern of trabecula in *Ccr3*$^{-/-}$ mice, while the trabecular separation was higher in *Ccr3*$^{-/-}$ mice compared to *Ccr3*$^{+/+}$ mice. Data represents mean values ± SD, n=16 per genotype. $^{**}p < 0.01$; *ns*, not significant.

Supplement 4.

List of included gene expression assays

Gene symbol	TaqMan gene expression assay (Applied Biosystem)	Gene name
Acp5/Trap	Mm00475698_m1	Acid phosphatase 5, tartrate resistant
Alpl/Alp	Mm00475834_m1	Alkaline phosphatase, liver/bone/kidney
Atp6v0d2	Mm01222963_m1	ATPase, H+ transporting, lysosomal V0 subunit D2
B2m	Mm00437762_m1	Beta-2 microglobulin
Bglap	Mm03413826_mH	Bone gamma carboxyglutamate protein
C-fos	Mm00487425_m1	FBJ osteosarcoma oncogene
Calcr	Mm00432282_m1	Calcitonin receptor
Ccl2	Mm00441242_m1	Chemokine (C-C motif) ligand 2
Ccl5	Mm01302427_m1	Chemokine (C-C motif) ligand 5
Ccr2	Mm00438270_m1	Chemokine (C-C motif) receptor 2
Ccr3	Mm01216172_m1	Chemokine (C-C motif) receptor 3
Ccr5	Mm01216171_m1	Chemokine (C-C motif) receptor 5
Clcn7	Mm00442400_m1	Chloride channel 7
Ctsk	Mm00484039_m1	Cathepsin K
Dcstamp	Mm01168058_m1	Dentrocyte expressed seven transmembrane protein
Fcer1g	Mm02343757_m1	Fc receptor, IgE, high affinity I, gamma polypeptide
Itgav	Mm00434486_m1	Integrin alpha V
Mmp9	Mm00600163_m1	Matrix metallopeptidase 9
NFATc1	Mm01265944_m1	Nuclear factor of activated T cells, cytoplasmic , calcineurin dependent 1
Oscar	Mm00558665_m1	Osteoclast associated receptor
Runx2	Mm00501584_m1	Runt related transcription factor 2
Sp7	Mm04209856_m1	Sp7 transcription factor 7
Spp1	Mm00436767_m1	Secreted phosphoprotein 1
Tnfrsf11a/Rank	Mm00437132_m1	Tumor necrosis factor receptor superfamily, member 11a, NFKB activator
Tnfsf11/Rankl	Mm00441906_m1	Tumor necrosis factor (ligand) superfamily, member 11
Tyrobp/DAP12	Mm00449152_m1	TYRO protein tyrosine kinase binding protein

Supplement 4. Table of analyzed genes in *Ccr3*$^{-/-}$ and *Ccr3*$^{+/+}$ BMM cultures and primary calvarial osteoblast cultures.
